# Coronavirus Disease (COVID-19) Caused by (SARS-CoV-2) Infections: A Real Challenge for Human Gut Microbiota

**DOI:** 10.3389/fcimb.2020.575559

**Published:** 2020-12-09

**Authors:** Dan-Cristian Vodnar, Laura Mitrea, Bernadette-Emoke Teleky, Katalin Szabo, Lavinia-Florina Călinoiu, Silvia-Amalia Nemeş, Gheorghe-Adrian Martău

**Affiliations:** ^1^ Institute of Life Sciences, University of Agricultural Sciences and Veterinary Medicine, Cluj-Napoca, Romania; ^2^ Faculty of Food Science and Technology, University of Agricultural Sciences and Veterinary Medicine, Cluj-Napoca, Romania

**Keywords:** metabolic disorders, nutrition, dysbiosis, gut microbiota, COVID-19, severe acute respiratory syndrome coronavirus 2, coronavirus

## Abstract

The current COVID-19 pandemic is a great challenge for worldwide researchers in the human microbiota area because the mechanisms and long-term effects of the infection at the GI level are not yet deeply understood. In the current review, scientific literature including original research articles, clinical studies, epidemiological reports, and review-type articles concerning human intestinal infection with SARS-CoV-2 and the possible consequences on the microbiota were reviewed. Moreover, the following aspects pertaining to COVID-19 have also been discussed: transmission, resistance in the human body, the impact of nutritional status in relation to the intestinal microbiota, and the impact of comorbid metabolic disorders such as inflammatory bowel disease (IBS), obesity, and type two diabetes (T2D). The articles investigated show that health, age, and nutritional status are associated with specific communities of bacterial species in the gut, which could influence the clinical course of COVID-19 infection. Fecal microbiota alterations were associated with fecal concentrations of SARS-CoV-2 and COVID-19 severity. Patients suffering from metabolic and gastrointestinal (GI) disorders are thought to be at a moderate-to-high risk of infection with SARS-CoV-2, indicating the direct implication of gut dysbiosis in COVID-19 severity. However, additional efforts are required to identify the initial GI symptoms of COVID-19 for possible early intervention.

## Introduction

The world is currently facing a major public health crisis due to the new coronavirus disease called COVID-19 caused by severe acute respiratory syndrome coronavirus 2 (SARS-CoV-2), which first emerged at the end of the year 2019 (Munster et al., 2020). The COVID-19 pandemic has placed the entire scientific community from all research fields on alert, considering that SARS-CoV-2 infected millions and killed hundreds of thousands of people in essentially every country in the world (WHO, 2020b). The pandemic situation put great emphasis on the use of mathematical calculations to be used as linear regression tools or even machine learning models to predict the number of cases (Parry et al., 2020; Syed et al., 2020). Up to September 4, 2020, the top three countries with the highest number of infected cases are represented by the US, Brazil, and India (Shah et al., 2020). A comprehensive list of pulmonary and extrapulmonary manifestations of the COVID-19 has been reported (Aziz et al., 2020; Gandhi et al., 2020; Johnson et al., 2020; Perisetti et al., 2020c). The pulmonary symptoms included fever, cough, chest tightness, fatigue, shortness of breath, hypoxemia, anosmia, nasal congestion, inflammatory storms, and viral pneumonia. The extrapulmonary manifestations were cardiac [cardiac arrhythmias, myocarditis, pericarditis, acute coronary syndrome (ACS), heart failure, cardiogenic shock, and cardiac arrest], neurological (headache, acute cerebrovascular disease, dizziness, and encephalopathy), hepatic [elevations in serum levels of alanine transaminase (ALT), aspartate transaminase (AST), and bilirubin, decreased levels of albumin], renal (acute kidney injury), ocular (chemosis, epiphora, and conjunctival congestion), dermatologic (erythematous rash, vesicular lesions, and urticaria), and gastrointestinal (GI) (Cha et al., 2020; Johnson et al., 2020; Jothimani et al., 2020). In addition, a systematic review and meta-analysis assessed the taste changes (dysgeusia) in COVID-19 patients (Aziz et al., 2020), whereas half of the patients (49.8%) had altered taste manifestation.

The most common GI symptoms included nausea, vomiting, diarrhea, and abdominal pain (Hamid et al., 2020; Li G. et al., 2020; Cavaliere et al., 2020; Wan et al., 2020; Wong et al., 2020; Jin et al., 2020). Clinical studies reported an increasing number of GI symptoms in patients with COVID-19, whereas diarrhea is the most common with an incidence rate between 2%-50% of cases, while the differences varies with age, various comorbidities, regions, lifestyle, dietary habits, etc. (D’amico et al., 2020; Zuo et al., 2020). However, the long-term consequences of SARS-CoV-2 infection in the GI tracts are not yet fully understood (Cheng et al., 2007; Ianiro et al., 2020).

The role of the intestinal microbiota in influencing lung diseases has been well articulated. It is also known that respiratory virus infections cause disturbances in the intestinal microbiota (Precup and Vodnar, 2019; Pan et al., 2020; Wan et al., 2020; Xiao et al., 2020; Zhang W. et al., 2020). Therefore, several studies have demonstrated the changes caused by COVID-19 infection in fecal microbiomes, precisely a gut dysbiosis characterized by opportunistic pathogens and depletion of beneficial commensals. This medical frame persisted even after clearance of SARS-CoV-2 (provided by throat swabs) and diminished respiratory symptoms. In addition, the baseline enrichment of *Coprobacillus*, *Clostridium ramosum*, and *Clostridium hathewayi* was associated with COVID-19 severity; there was an inverse correlation between the abundance of *Faecalibacterium prausnitzii* and disease severity. *Bacteroides* subspecies, which downregulates expression of ACE2 in the murine gut, correlated inversely with SARS-CoV-2 load in fecal samples from patients (Amirian, 2020; Zuo et al., 2020).

It is well known the dysbiosis association with the development of obesity, metabolic syndrome, inflammatory bowel disease (IBD), irritable bowel syndrome (IBS), type 2 diabetes (T2D), and other metabolic disorders (Harley and Karp, 2012; Ahsan et al., 2013; Buie et al., 2019; Dhar and Mohanty, 2020; Wang P. X. et al., 2020). Dysbiosis is characterized by low microbial diversity including a reduced abundance of *Bifidobacterium* spp., *Lactobacillus* spp., and *Faecalibacterium prausnitzii* (Dhar and Mohanty, 2020). Therefore, people suffering from these disorders are much more prone to viral and intestinal SARS-CoV-2 infections, mainly because of the existing disturbances within their gut microbiota (Liu and Lou, 2020). In addition, the intake of medications is the second most important factor which strongly disturbs the gut microbiota (Dhar and Mohanty, 2020). Medications commonly used to treat chronic autoinflammatory conditions are associated with higher rates of serious viral and bacterial infections, including influenza and pneumonia (Brenner et al., 2020). Moreover, the diversity of intestinal microbiota is low in old age, and COVID-19 has been observed to be fatal in elderly patients (Li Q. et al., 2020; Rothan and Byrareddy, 2020; Wang L. F. et al., 2020).

Thus, of major worldwide importance is to envisage a role for the intestinal microbiota in the manifestation of this disease. Nutrition/diet, environmental factors, and genetics play an important role in shaping the intestinal microbial population. Improving the profile of the intestinal microbiota may be a prophylactic measure to minimize the impact of COVID-19 in elderly and immunodeficient patients (Dhar and Mohanty, 2020). The COVID-19 pandemic constitutes a new challenge in terms of the nutritional status of patients worldwide. Gastroenterologists and nutritionists must put their efforts together in maintaining the patients’ health state by indicating the most appropriate nutritional program to stimulate the innate immune system to face these viral challenges.

The aim of the current review was to assess and understand the COVID-19 challenge for human microbiota, in case of comorbid metabolic disorders such as IBS, obesity, and T2D, while underlying the impact of nutrition in relation to the intestinal microbiota. Moreover, the transmission and resistance in the human body of the virus, in the GI context, have also been reviewed and discussed.

### Methods of Literature Selection

**Figure 1:**
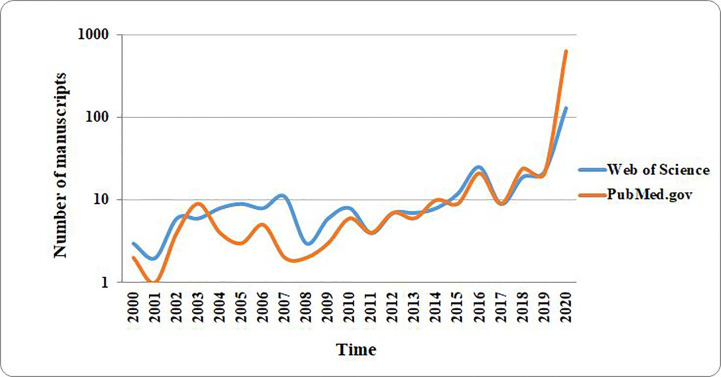
The distribution of number of research papers published on PubMed and Web of Science over the past two decades. The results plotted in the graph were obtained by searching the cited databases for articles published over the past two decades. The search keywords were as follows: [(COVID) OR (coronavirus) OR (COVID-19) OR (2019-nCoV) OR (SARS-CoV-2)] AND [(gut) OR (microbiota) OR (diabetes) OR (obesity)].

For the present paper, a literature selection including original research articles, clinical studies, epidemiological reports, and review-type articles was applied. The electronic databases from Medline (PubMed interface) (PM), and Web of Science Core Collection (WOS) were searched using the keywords [(COVID) OR (coronavirus) OR (COVID-19) OR (2019-nCoV) OR (SARS-CoV-2)] AND [(gut) OR (microbiota) OR (diabetes) OR (obesity)]. A significantly high number of articles were found for the period between 2000 and the present time (i.e., June 9, 2020) (**Figure 1**). Studies on COVID-19 have led to a large number of publications in recent months (December 2019 to June 2020) associated with the global pandemic. Over 500 publications in the last month were related to the topic “[(COVID) OR (coronavirus) OR (COVID-19) OR (2019-nCoV) OR (SARS-CoV-2)] AND [(Gut) OR (microbiota) OR (diabetes) OR (obesity)]”. In the last two decades (2000–2020), a total of 1,096 works were published considering these topics, from which 151 publications were review articles. From this interval, 88.46% of the publications included in WOS were open access, while those covered by PM, as open access, were about 60.97%.

All the manuscripts abstracts were independently screened by applying inclusion/exclusion criteria (**Table 1**). There were evaluated the retrieved full-text articles applying the same inclusion and exclusion criteria that were used for the abstract selection. Any disagreements during the selection process were discussed among all the authors and unit consensus was reached.

**Table 1:** Inclusion and exclusion criteria.

Inclusion criteria	Exclusion criteria
1. Studies published in the last twenty years, from 2000 to June 2020 were included in the search; 2. English language; 3. Human and animal (rodent) studies; 4. Studies that report the impact of diet, obesity, diabetes, on the presence COVID in the intestinal ecosystem or studies comparing the long-term and short-term impact of diets on the intestinal microbiota or studies reporting the metabolite’s signature on this COVID; 5. Studies that report the correlation between host health and the presence of COVID virus in the intestinal ecosystem.	1. Articles that select COVID virus from other animals (not rodents); 2. Data sets that did not provide information on dietary, dietary, intervention, and nutritional properties associated with the presence of COVID in the gut; 3. Failure to provide data for the presence of COVID in the intestinal ecosystem.

## Current Knowledge of SARS-COV-2 Transmission and Pathogenicity

**Figure 2:**
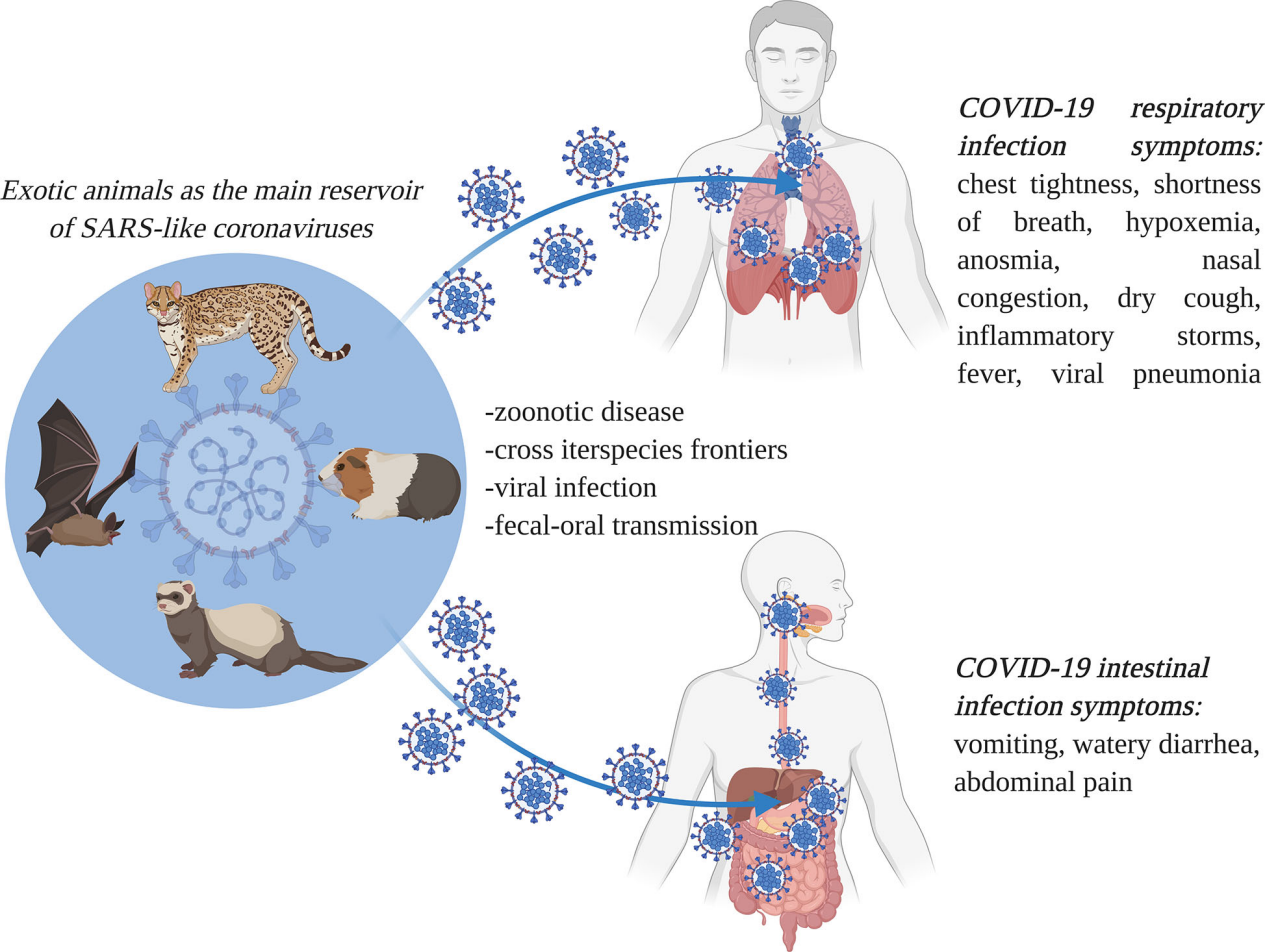
Interspecies severe acute respiratory syndrome coronavirus 2 (SARS-CoV-2) (causal organism for the coronavirus disease (COVID-19)) transmission routes, and the associated respiratory and gastrointestinal symptoms. SARS-CoV-2 has extreme transmissibility, and it successfully jumps from animal to human hosts, causing major outbreaks of COVID-19, an emerging zoonotic disease. It causes both respiratory and gastrointestinal symptoms (image created using BioRender application https://app.biorender.com).

It has been pointed out by various researchers that coronaviruses represent a group of viral entities which can traverse interspecies frontiers (Decaro et al., 2020; Lam et al., 2020), and exotic wild animals represent very important reservoirs in spreading the infection (Cheng et al., 2007; Rothan and Byrareddy, 2020; Zhou et al., 2020). As it is illustrated in **Figure 2**, SARS-like coronaviruses can successfully jump from animal to human hosts, due to their efficient adaptation and transmission mechanisms (Mackenzie and Smith, 2020; Wang L. F. et al., 2020). This feature is responsible for the COVID-19 pandemic, which is one of the major worldwide outbreaks of emerging zoonotic diseases in the last 25 years.

In general, pathogens such as coronaviruses are considered to be the major agents of emerging respiratory disease outbreaks (Preckel et al., 2020). In addition, Wolfel et al. reported a detailed virological analysis of nine cases of COVID-19 that provides proof of active virus replication in tissues of the upper respiratory tract (Wolfel et al., 2020). The SARS-CoV-2 entity measures about 60–140 nm in diameter and usually presents with an elliptic and often pleomorphic shape. The genetic material consists of a large single-stranded positive RNA (+ssRNA) and can be isolated from different animal reservoirs. This aspect allows the SARS-like coronaviruses to cross the species barrier and adapt to human bodies. The RNA-based genome consists of 29,891 nucleotides, encoding about 9,860 amino acids. Genetic analysis reveals that SARS-CoV-2 may have evolved from a particular strain of a coronavirus found in bats (Cascella et al., 2020; Walls et al., 2020). This hypothesis is supported by the observation that the genomic fingerprint of the human SARS-CoV2 is similar to that of coronaviruses found in wild animals. For instance, Chan et al. performed a genomic analysis of a SARS-CoV-2 isolate obtained from a patient presenting with atypical pneumonia after visiting Wuhan and indicated that the genetic profile showed 89% nucleotide identity with that of the bat SARS-like CoVZXC21 (Chan et al., 2020a).

**Figure 3:**
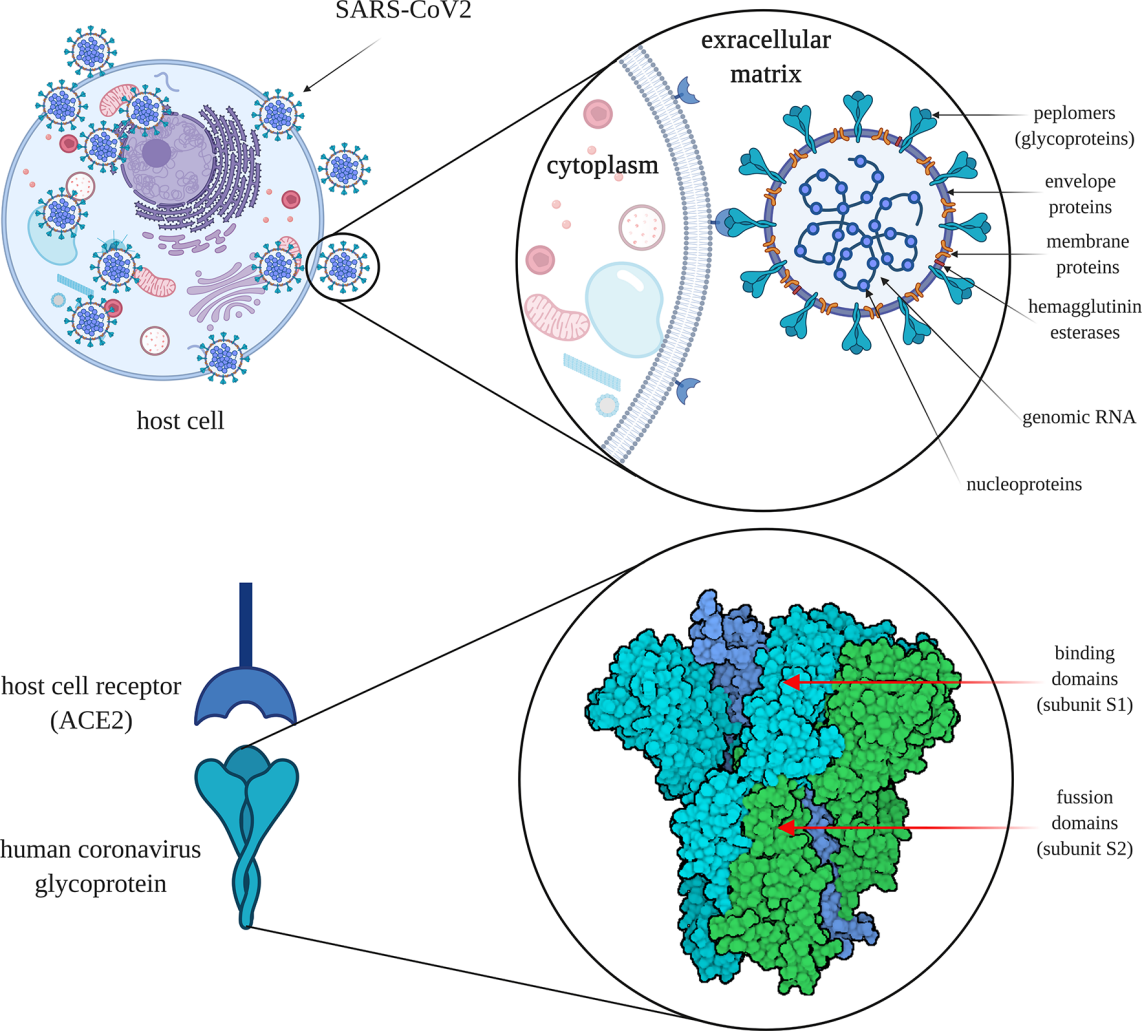
The severe acute respiratory syndrome coronavirus 2 (SARS-CoV-2) cell infection mechanism. SARS-CoV-2 is surrounded by spiky glycoproteins, also defined as peplomers. The glycoproteins consist of two functional subunits, S1 and S2. The S1 subunit is responsible for host cell receptor binding and stabilization of the viral entity, while the S2 subunit facilitates the fusion of the viral and cellular membranes. After invading the human body, SARS-CoV-2 binds to angiotensin converting enzyme 2 (ACE2), which is found on the exterior surfaces of multiple types of cells. ACE2 - angiotensin converting enzyme 2 (image created using BioRender application https://app.biorender.com).

Under the electron microscope, the surface of SARS-CoV-2 appears to have a crown-like layer made of spiky projecting glycoproteins, which are defined as peplomers; the viral particles range in size from 80–160 nM and include a 27–32 kb genome of positive polarity (Cascella et al., 2020; Sahin, 2020). The pathogenicity and virulence mechanisms of SARS-CoV-2 are linked to the external layer of glycoproteins, which are capable of blocking the innate immune response of the host (Lei et al., 2018; Cascella et al., 2020). These spiky glycoproteins consist of two functional subunits, namely S1 and S2; the S1 subunit is responsible for host cell receptor binding and stabilization of the viral entity, while the S2 subunit helps in the fusion of the viral envelope with the host cell membrane (**Figure 3**) (Walls et al., 2020).

The molecular attachment between the viral entity and the human host cell is an important characteristic of SARS-like coronaviruses (Ding and Liang, 2020). After invading the human body, SARS-CoV-2 binds to angiotensin-converting enzyme 2 (ACE2) protein, which is found on the membranes of multiple types of cells (alveolar cells, myocardium, pancreas, enterocytes, spleen, thymus, bone marrow, liver, kidney, and brain) (Hamming et al., 2004; Hoffmann et al., 2020; Jia et al., 2020; Muniyappa and Gubbi, 2020; Perisetti et al., 2020c). Actually, SARS-CoV-2 presents an elevated binding affinity to the ACE2 receptors. The virus is internalized by the cells through endocytosis from where it generates viral RNA and viral-specific proteins. When the viral entity is replicated, it transpasses the cells through secretions. Within the GI tract, the viral secretions are followed by the release of the cytokines, which in turn are responsible for the associated GI manifestations (Dahiya et al., 2020). Still, it remains unknown how SARS-CoV-2 enters the GI tract and survives the extreme pH medium of the stomach (Ng and Tilg, 2020). Moreover, through body fluids (e.g., saliva, mucus, blood, feces, sputum, tears, semen), the virus can be transmitted from human-to-human (Mohseni et al., 2020). In addition, the transmission mechanism of SARS-CoV-2 is similar in the cases of asymptomatic patients too, so the contamination risk of medical personnel or the close persons of infected ones is extremely high (Yu and Yang, 2020).

In the case of SARS-CoV-2, the interaction of the virus with the host cell and implicitly the host infection is related to the general health status of the host and its innate immune response. For patients suffering from enteric viral infections or other inflammatory conditions, the immune response to SARS-CoV-2 may be significantly altered because of differences in cytokine profiles compared to those in healthy individuals (Ding and Liang, 2020). There is a great diversity in the cytokine profile regarding the interleukins (ILs) (IL-2, IL-6, IL-10, IL-17, IL-22, and IL-23) in healthy persons based on age, sex, and general health status. Clinical reports of patients affected by COVID-19 revealed the presence of a large number of T lymphocytes and mononuclear macrophages which were in the activated state. The activation of these immune cells results in the expression of a number of cytokines such as interleukin-6 (IL-6), further leading to cytokine storms and severe inflammatory responses in the lungs, as well as in the other organs (Xu et al., 2020). The levels of cytokines, especially those of IL-6 and IL-23, are much lower in healthy persons than in virus-infected patients (Perisetti et al., 2020b; Zhang C. et al., 2020).

In most of the cases of SARS-CoV-2 infected patients, respiratory symptoms were among the first reported (Amirian, 2020). Still, since the virus can replicate in both the respiratory and GI tract, it can be easily transmitted through the fecal-oral route (Hussain N. et al., 2020; Kopel et al., 2020). Moreover, it is supposed that the virus can be re-transmitted through feces by aerosolization of the viral-containing droplets, but the supposition is not yet confirmed (Holshue et al., 2020; Kopel et al., 2020).

An important aspect regarding the transmission of the COVID-19 is represented by the risk factors and how they are managed by the medical staff particularly. Special attention is paid to the prevention methods by medical staff treating patients infected with COVID-19 and should be paid also by doctors and nurses who may come into contact with the infection risk factors (blood, body fluids), for example, the department of endoscopy (Perisetti et al., 2019; Johnston et al., 2019). Endoscopy departments are exposed to a significant risk of distribution of the respiratory disease that can be spread by the air through oral and fecal material that can be aspirated by endoscopes (Repici et al., 2020). As Perisetti et al. are explaining, the endoscope is in direct contact with the intestinal flora, which is a major vector of viral transmission, increasing the risk of infection for the endoscopists, nurses, other endoscopy workers, and also for future patients (Perisetti et al., 2020a). Unfortunately, at this time there is no method or protocol for disinfecting the endoscope properly for assuring maximum safety and protection for patients, which makes duodenoscope-associated infection the most common hospital contamination source (Rahman et al., 2019).

## Resistance in the Human Body: How is Human Microbiota Affected by COVID-19

As per gastroenterologists, human-to-human transmission of SARS-CoV-2 is possible by airborne respiratory droplets and by the fecal-oral route, raising the possibility of GI manifestations of the disease (D’amico et al., 2020; Ding and Liang, 2020; Gu et al., 2020).

SARS-CoV-2 affects both the respiratory and GI tracts, and symptoms such as watery diarrhea are associated with a prolonged span of the disease and viral transmission in COVID-19 (Galanopoulos et al., 2020; Wei et al., 2020). The worldwide scenario presents that the initial symptoms of the SARS-CoV-2 respiratory and intestinal infections appear in approximately 5.2 days, while the time interval from symptom onset to death ranges from 6 to 41 days, with a mean of about 14 days. This timeline is closely related to the patient’s health status and age (Li Q. et al., 2020; Rothan and Byrareddy, 2020; Wang L. F. et al., 2020).

Fecal samples of SARS-CoV-2 positive patients tested by RT-PCR have confirmed viral presence in the intestines (RNA-based genome), which is an additional factor requiring attention and dedicated management (Chen et al., 2020; Tang et al., 2020). The presence of the SARS-CoV-2 viral genome in feces is associated with the presence of the virus unit within the GI tract (Pal et al., 2020). Furthermore, Park et al. have reported in clinical trials that the virus can be present in fecal samples for up to 50 days (Park et al., 2020). Recent clinical studies point out that diarrhea manifests in 2% to 50% of COVID-19 cases, and this symptom may appear in the absence of, may precede, or may accompany respiratory symptoms (D’amico et al., 2020; Patel et al., 2020; Perisetti et al., 2020b). For example, in the first case of COVID-19 infection reported in the United States of America, the patient presented with respiratory symptoms, nausea, vomiting, and diarrhea. RT-PCR analysis indicated the presence of SARS-CoV-2 in both the nasopharyngeal and oropharyngeal mucosa, and in diarrhea specimens, while the serum was negative for the virus (Ding and Liang, 2020; Holshue et al., 2020). During clinical episodes of acute and/or severe diarrhea, the human GI tract is highly dehydrated and a high level of pathogen colonization and infestation is observed (Mitrea et al., 2017; Da Cruz Gouveia et al., 2020). Severe diarrheal episodes cause a strong imbalance and dysbiosis within the intestinal ecosystem and significantly affect electrolyte availability, leading to a rapid weakening of general health (Guyton and Hall, 2010). In most of the cases, COVID-19 patients have prescribed antibiotics like fluoroquinolones and cephalosporins to exclude any secondary bacterial infections, and this fact generates diarrhea as an adverse effect. Also, drugs with antiviral activity such as ritonavir-lopinavir, hydroxychloroquine, or remdesivir are administered to COVID-19 patients, and diarrhea appears as a side effect of these treatments (Perisetti et al., 2020b). Moreover, as Perisetti et al. (2020b) highlight in their letter, diarrhea is common in patients suffering from GI diseases like IBD, where ACE2 receptors are overexpressed and increase the risk of diarrhea in patients diagnosed with COVID-19. For example, in a clinical report of Cavaliere et al. is mentioned that six patients hospitalized with COVID-19 pulmonary symptoms (fever, shortness of breath requiring oxygen), presented also upper GI bleeding (GIB) that manifested through hematemesis or melena. In this clinical report, the GI bleeding was assumed to be correlated with ulcer, and also with the COVID-19-associated coagulopathy (Cavaliere et al., 2020). Another clinical report (Gadiparthi et al., 2020) shows that SARS-CoV-2 respiratory symptoms (e.g., acute hypoxic respiratory failure, shortness in breath, flu-like symptoms) were accompanied by GIB manifested through melena in three patients of different ages and genders. It was also observed that GIB caused acute hemoglobin decrease that required urgent transfusion for all three patients in order to be stabilized (Gadiparthi et al., 2020). Among the common GI symptoms observed in patients diagnosed with COVID-19, acute pancreatitis was reported in some patients from a clinical study of Inamdar et al. where patients manifested upper abdominal pain (Inamdar et al., 2020).

SARS-CoV-2 survives successfully in the gut of asymptomatic persons. For example, the SARS-CoV-2 viral RNA fingerprint was found in stool samples of an asymptomatic child whose parents were found to be SARS-CoV-2 negative at a 2-week interval (Amirian, 2020; Tang et al., 2020). Contrary to the above, in one case report, an asymptomatic infant was tested and found to be negative for viral RNA in stool specimens, even though he had been in close contact with his parents who tested positive for the virus (Amirian, 2020; Kam et al., 2020). As mentioned in **Table 2**, multiple clinical reports from around the world prove that SARS-CoV-2-infected individuals present with GI symptoms in addition to respiratory symptoms.

**Table 2:** Clinical reports of corona virus disease (COVID-19) cases with gastrointestinal symptoms.

Gastrointestinal symptoms	Geographic location	Definition of positive result	Relevant specimen type	Number of positive patients/total number tested (%)	References
Authors reported that a subset of patients (n = 5) were treated with antivirals, and of those, four developed GI symptoms (Treatment is likely a confounding factor)	Singapore	DVR: ORF1ab, spike, and nucleoprotein gene; Ct-value <40	Fecal samples	4/8 (50)	(Young et al., 2020)
Three patients with diarrhea were positive for viral RNA in stool; transient GI symptoms were also noted in patients treated with antiviral drugs	United States	DVR: details not specified (appendix of laboratory methods unavailable at time of writing)	Fecal samples	7/10 (70)	(Holshue et al., 2020)
–	Shanghai, China	Laboratory test data were abstracted from medical records to determine whether samples were positive for viral RNA through RT-PCR	Fecal samples	54/66 (82)	(Ling et al., 2020)
–	Guangzhou, China	DVR: ORF1ab and nucleoprotein gene; Ct-value not specified (Authors stated that positives were defined as one or both primer/probe sets providing a “reliable signal”)	Anal swabs ^e^	11/28 (39)	(Chen et al., 2020)
Two patients with diarrhea were negative for viral RNA in stool, but the authors pointed out that the timing of fecal specimen collection was after diarrhea had subsided	Guangdong, China	DVR: RdRp (RNA dependent RNA polymerase) and spike genes; CT value not directly specified	Fecal samples	0/7 (0)	(Chan et al., 2020b)
No GI symptoms; Patient was virtually asymptomatic	Kallang, Singapore	DVR: ORF1ab and nucleoprotein gene; all Ct-values reported as positive <40, but the threshold was not directly specified	Fecal samples	1/1	(Kam et al., 2020)
Authors stated that the presence of GI symptoms was not associated with viral RNA presence in fecal samples	Zhuhai, China	DVR: RdRp, nucleoprotein, and membrane genes; Ct-value not directly specified	Fecal samples	41/74 (55)	(Wu Y. et al., 2020)
Case was asymptomatic	Zhoushan, China	DVR: ORF1ab and nucleoprotein gene; authors reported that Ct-values for results considered positive were all <40, but the threshold was not directly specified	Fecal samples	1/3 (33)	(Tang et al., 2020)
A subset of patients (approx. 40%) who tested positive for viral RNA in fecal samples had diarrhea; a very small number also exhibited GI bleeding	Zhuhai, China	DVR: ORF1ab and nucleoprotein gene; Ct-value <37; Viral nucleocapsid staining in biopsy tissue collected through endoscopy	Fecal samples	39/73 (53)	(Xiao et al., 2020)
No diarrhea noted	Shanghai and Qingdao, China	DVR: ORF1ab and nucleoprotein gene; Ct-value <35	Fecal samples	Five patients whose samples initially tested positive were retested and remained positive on follow-up test (day 18–30)	(Cai J. et al., 2020)
Four patients initially negative for virus from anal swabs (and from oral swabs) on the first day of sampling became positive from anal swabs on the fifth day. Two other patients were positive on the first day on oral swabs, but negative on anal swabs.	Wuhan, China	DVR: spike gene; Ct-value <40	Anal swabs ^a^	First day of sampling: 4/16 (25) Fifth day of sampling: 6/16 (38)	(Zhang W. et al., 2020)
Some serial test results on fecal samples were available for six patients	Jinhua, China	DVR; details not specified	Fecal samples	5/14 (36)	(Zhang J. et al., 2020)
	Hubei, Shandong, and Beijing, China	DVR: ORF1ab; Ct-value <40; Fecal culture conducted on four samples with high copy numbers	Fecal samples	44/153 (29)	(Wang W. et al., 2020)

GI, gastrointestinal; DVR, Detection of viral RNA; ORF, Open reading frame; RT-PCR, Reverse transcriptase polymerase chain reaction; RdPp, RNA-dependent RNA polymerase.

a - Anal swabs may not be directly relevant as stool specimens to the topic under review for several reasons (e.g., differences in cell content), but are presented to provide a more comprehensive perspective.

**Figure 4:**
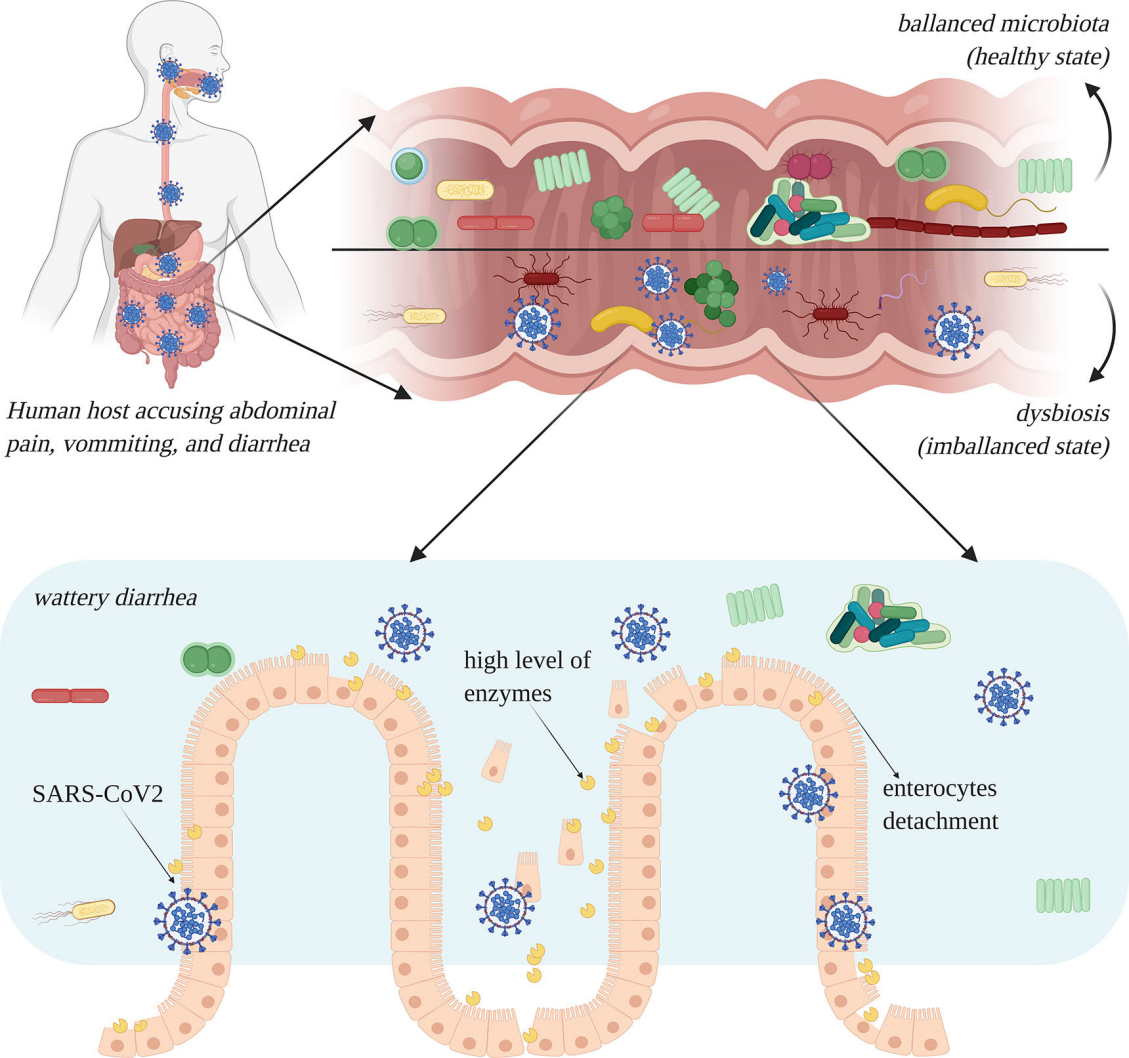
Intestinal infection with severe acute respiratory syndrome coronavirus 2 (SARS-CoV-2) leads to dysbiosis, especially due to the occurrence of diarrhea. Enterocytes are damaged and show a high level of overexpression of enzymes which lead to rapid cell degradation (image created using BioRender application https://app.biorender.com).

In an intestinal infection with SARS-like viruses, the GI mucosa may be seriously damaged, leading to cytopathic alterations that spread through the cell monolayers, causing cell detachment within 24 to 48 h (Cheng et al., 2007). In the case of a SARS-CoV-2 intestinal infection, the functions of mature enterocytes are derailed; several enzymes are highly overexpressed in atypical areas, which may lead to malfunctions or irreversible damage in enterocytes (**Figure 4**) (Ding and Liang, 2020). Lamers and co-authors reported that after 60 h of SARS-CoV-2 infection, enterocyte apoptosis is perspicuously obvious (Lamers et al., 2020).

A recent correspondence related to the impact of COVID-19 on gut microflora shows that the National Health Commission and the National Administration of Traditional Chinese Medicine have recommended the administration of probiotics to COVID-19 patients (dated end of February 2020) (Kam et al., 2020). One of the main reasons for this national measure is related to the fact that up to 70% of COVID-19 patients were administered antibiotics, so the susceptibility to subsequent intestinal infections was highly increased (Guan et al., 2020). Mak and group highlighted that the use of probiotics in SARS-CoV2 infection is unlikely to have a direct effect, especially because most of the symptoms in COVID-19 patients are respiratory (Mak et al., 2020). Two meta-analyses have reported modest efficacy of probiotic administration in reducing the frequency and span of respiratory infections. The same research group has suggested that the use of common probiotics is not recommended for the treatment of COVID-19 until the pathogenesis of SARS-CoV-2 is deeply understood along with its impact on the gut microbiota (Kam et al., 2020). On the contrary, other research studies point out that the administration of probiotics and nutraceuticals has a supportive role in enhancing the immune response and is effective in the prevention of viral infections in general (Kang et al., 2013; Mousa, 2017; Gao et al., 2020; Jayawardena et al., 2020; McCarty and Dinicolantonio, 2020).

## Metabolic Disorders as Risk Factors for COVID-19

Microbial gene products have an important effect on the metabolism and health status of the host, and therefore, a compositional change in the microbiota and/or an abnormality in the interactions between the host and the commensal microbiota leads to dysbiosis. Furthermore, a dysbiosis in microbiota can lead to the loss of regulatory immune effects on the gut mucosa, and this condition is associated with several inflammatory and immune-mediated diseases (Cheng and Ning, 2019).

SARS-CoV-2 represents a double challenge for people with metabolic diseases. T2D, bowel diseases, and obesity have been reported to be important risk factors impacting the severity of COVID-19. For this reason, patients need to pay special attention to the preliminary symptoms of this disease to avoid severe complications (Bilal et al., 2020; Dhar and Mohanty, 2020).

### Impact of Nutrition on SARS-CoV-2 Infection

Multiple clinical studies have shown that worldwide deaths and severe complications in COVID-19 cases were reported among elderly people with a medical history of chronic diseases (cardiovascular, liver, and kidney diseases, and cancer) (Caccialanza et al., 2020; Huang et al., 2020; Wang L. F. et al., 2020; Guo, 2020). The nutritional status of all COVID-19 patients should be evaluated at the time of hospital admission, but special attention must be paid to those with an increased risk both of infection (elderly patients and patients with chronic diseases) as well as of malnutrition. For example, patients with a nutritional risk should be closely observed and oral nutritional supplements must be administered to increase protein intake in order to support the immune system (Brugliera et al., 2020). Moreover, it was observed that patients with COVID-19 exhibited a protein deficit (i.e., prealbumin), even though they were not at risk of malnutrition before the infection (Caccialanza et al., 2020; Wu C. et al., 2020). The administration of nutritional supplements consisting of multivitamins and minerals maintains the host health state, and because of the antioxidant properties of the supplements, the severity of viral infections is significantly diminished (Weger-Lucarelli et al., 2019; Caccialanza et al., 2020; Galanakis, 2020).

Nutrition-related disorders or metabolic diseases lead to chronic health disorders, which place the affected persons at increased risk of infection with SARS-CoV-2. Because of prolonged life expectancy, multiple metabolic disorders (such as obesity, IBS, T2D, and nonalcoholic fatty liver disease) have become important health problems worldwide, and constitute a source of socioeconomic burden; there is a need for an overall scenario of prevention and management of such conditions (Butler and Barrientos, 2020; Deng et al., 2020; Lidoriki et al., 2020; Marhl et al., 2020; Nie et al., 2020). Besides, lifestyle habits such as the intake of unhealthy food products and maintaining unwholesome diets lead to susceptibility to COVID-19 and difficulties in recovery. Thus, worldwide nutritionists and gastroenterologists recommend that people at a higher risk (older and chronic disease-affected individuals) must abstain from unhealthy eating habits and should try to consume more unprocessed food, whole grains, vegetables, and unsaturated fats to boost the immune system and induce protection against viral infections (Butler and Barrientos, 2020; Schuttelaar-Partners, 2020).

#### Gastrointestinal Diseases

Some of the most common GI diseases like IBS, IBD, GIB, and cirrhosis, are associated with intestinal dysbiosis (Collins et al., 2012). These diseases have been reported to increase the vulnerability of affected persons to SARS-CoV-2 infections (Lagana et al., 2020; Mao et al., 2020).

IBS has a considerably high prevalence, globally of 11% (Lovell and Ford, 2012), with main features of impaired bowel habit, and persistent or recurrent abdominal pains (Carco et al., 2020). IBD comprising ulcerative and Crohn’s disease is a chronic inflammatory disorder of the GI tract, and in developing countries has an ascending prevalence (Zuo and Ng, 2018). The terminal phase of chronic liver disease is cirrhosis which accounts for the main cause of death in the West and has several causes like alcohol abuse, chronic viral infection, or nonalcoholic fatty liver disease (Schwope et al., 2020). GIB especially upper GIB is a frequently seen problem in elderly patients with a case fatality percentage between 5% and 14% (Valkhoff et al., 2012). It is well accepted that genetic, immune, and environmental factors are involved in GI diseases development. Modifiable factors include dietary habits that influence the gut microbiome, and these factors play an important role in disease development.

Therefore, the main question is: Does the COVID-19 pandemic impact the management of dysbiosis, including that of patients with GI diseases? According to the literature, patients suffering from bowel-related disorders are thought to be at moderate to high risk of infection with SARS-CoV-2 (Iacucci et al., 2020). Numerous case studies (D’amico et al., 2020; Han et al., 2020; Hindson, 2020; Pan et al., 2020; Wu Y. et al., 2020; Xiao et al., 2020) have found the presence of SARS-CoV-2 RNA in fecal samples of COVID-19 patients, including in those of several patients with a negative result in the upper respiratory tract test. In a case study by Wu and group (Wu Y. et al., 2020), the viral RNA result was positive in fecal samples for a mean of 11 days (and up to 5 weeks) after throat swabs were confirmed as negative for the virus (Wu Y. et al., 2020). This finding may suggest that the virus is constantly replicating in the GI tract of the patient and that fecal-oral transmission may occur after viral confirmation in the respiratory tract. In the same study, the presence of GI symptoms was not associated with viral RNA positivity in fecal samples (Wu Y. et al., 2020).

Over the past decades, numerous studies have repeatedly identified imbalanced gut microbiota, in patients suffering from IBD. A recent study involving IBD patients found that older age, increased number of comorbidities, and systemic corticosteroid use are strong risk factors for adverse COVID-19 outcomes (Brenner et al., 2020). In patients with COVID-19 found in critical state digestive manifestations occur in 36%–50.5% (Gulen and Satar, 2020). In cirrhosis GI wall thickening or the misuse of alcohol is indistinctly linked to the gut microbiota (Bajaj, 2019; Schwope et al., 2020). SARS-CoV-2 related liver damage reported in infected patients, is mostly due to enhanced systemic inflammation and immune immobility, causing profound immunological dysfunctions or even increased mortality rate (Rela et al., 2020; Iavarone et al., 2020).

The COVID-19 pandemic has resulted in an increased need for medical staff for the management of the affected patients (Danese et al., 2020; Gralnek et al., 2020). This has adversely affected the diagnosis of IBD patients, due to reduced availability of staff to perform endoscopic testing. Therefore, patients with IBD have been advised to follow general public health recommendations defined by the WHO. The general approach outlined by the British Society of Gastroenterology, the International Organization for the Study of Inflammatory Bowel Disease, the European Crohn’s and Colitis Organization, and the Crohn’s and Colitis Foundation of America is to diminish interaction with healthcare sites by canceling all non-emergency endoscopic testing, as such sites are perceived as contamination sites for both patients and medical personnel (Mao et al., 2020). Patients with IBD have symptoms which are generally chronic, in contrast to the acute presentation of symptoms in COVID-19 cases. Also, the effect of COVID-19 on patients suffering from GIB had adverse effects and outcomes, with prolonged hospitalization, worse laboratory findings, and unattended endoscopy (Kim et al., 2020).

Whether the viral RNA detected in fecal samples is infectious, is as yet unclear (Wolfel et al., 2020). Considering the generally accepted model of the so-called “gut-lung axis” in influenza infections (Bradley et al., 2019), involving the modulation of lung injury by the gut microbiome and gut wall permeability, the COVID-19 infection may induce a systemic inflammatory response via the gut epithelial passage (Feldstein et al., 2020).

#### Obesity

Obesity currently affects over 650 million people and is defined based on a body mass index (BMI) value of over 30 kg/m^2^. Approximately, 45% of adults worldwide are obese. Obesity defined by excess body weight presents significant risks for many chronic diseases, particularly cardiovascular disease, cancer, nonalcoholic fatty liver disease, and T2D (Nie et al., 2020). Obesity disrupts gut homeostasis with an increased *Firmicutes/Bacteroidetes* ratio, which in turn promotes adiposity. Obesity has a detrimental effect on insulin sensitivity and inflammation and additionally has a negative impact on behavior. Some studies have shown that obese patients have diminished diversity in gut bacteria, while others indicate that gut dysbiosis can be the outcome and enhancer of obesity (Buie et al., 2019). Gut microbiota impairment can be used to discriminate between obese and lean people with a ~90% precision (Walters et al., 2014). Metabolic disorders such as obesity and T2D are some of the disorders related to imbalanced gut microbiota (dysbiosis) (Ley et al., 2005; Amabebe et al., 2020).

With the rapid increase in obesity worldwide, the effect of this condition on transmissible diseases is being increasingly recognized (Honce and Schultz-Cherry, 2019). Several studies have shown that in influenza A H1N1 virus infections, obesity is a significant risk factor for the development of a more severe form of the disease, and may lead to an increased dispersion period (Honce and Schultz-Cherry, 2019; Moser et al., 2019).

The disease severity of COVID-19 in obese patients is mostly due to the consistent presence of severe insulin resistance (due to leptin resistance), imbalanced activity in the renin-angiotensin-aldosterone system (Lavie et al., 2020), hypoventilation, and restrictive pulmonary disease (Chiappetta et al., 2020). Insulin resistance has the eventual outcome of hyperglycemia and T2D. The expression of ACE2 in the adipose tissue is thought to be greater than that in the lung tissue, which leads obese individuals to be more susceptible to COVID-19 (Lavie et al., 2020). Adipose tissue secretes inflammatory cytokines (tumor necrosis factor (TNF)α, interleukin (IL)-10, IL-6, IL-1, and leptin), leading to low-grade systemic inflammation. In obesity, chronic inflammation is associated with unnatural cytokine production and elevated acute-phase reactants. This excessive cytokine generation (cytokine storm) can cause inadequate viral replication control and extended pro-inflammatory responses. The subsequent outcome is accelerated disease progression which may lead to multi-organ failure (Chiappetta et al., 2020). The characteristics of inflammation are ischemia (insufficient blood flow) and hypoxia (insufficient oxygen levels in the blood and adipose tissue).

A study conducted in Shenzhen, China including 383 patients with COVID-19 revealed that obesity significantly increased the severity of disease progression (Cai Q. et al., 2020). Among underweight patients, no severe cases were reported, while severe cases were observed exclusively in overweight or obese patients (especially in men) (Cai Q. et al., 2020). A study conducted in New York, United States, reported that obesity presented an epidemiologic risk factor for higher morbidity rates among individuals aged <60 years (Kass et al., 2020; Lighter et al., 2020). Zhang and group (Zhang C. et al., 2020) demonstrated that obesity contributed to the death of young COVID-19 patients with enhanced inflammation and enlarged lymph nodes. In deceased patients, the expression of inflammatory factors (higher IL-10, TNF-α, and CRP values), myocardial damage (higher B-type natriuretic peptide (BNP) and high-sensitivity cardiac troponin I (hs-cTnI)), and secondary fibrinolysis (increased D-dimer level with abnormal coagulation) were observed (Zhang F. et al., 2020).

Additionally, the treatment of obese patients presents several “mechanistic” issues, such as the requirement of bariatric beds, difficulties in performing diagnostic imaging (equipment with a weight limit), decreased airway flow, and difficulty in intubation (Michalakis and Ilias, 2020). In addition to older age, the occurrence of severe obesity (BMI > 40 kg/m^2^), T2D, and hypertension increase the morbidity and mortality rate of patients infected with SARS-CoV-2 (Muniyappa and Gubbi, 2020).

#### Type 2 Diabetes

The worldwide scenario presents a high incidence of T2D, which is distinguished by skeletal muscle, adipose tissue, and liver insulin resistance, resulting from incapacitated insulin secretion through pancreatic β-cells (Esser et al., 2014; Defronzo et al., 2015).

As predicted by the International Diabetes Federation, there will be approximately 592 million cases of T2D by the year 2035 (Ebrahimzadeh Leylabadlo et al., 2020). Dysbiosis in T2D affects the onset and maintenance of insulin resistance, and metagenomics analyses have indicated that T2Dpatients manifest diminished gut microbiota and dysbiosis in comparison with IBS (Gonzalez-Arancibia et al., 2019). The enrichment of harmful bacterial species and metabolic mediators is an additional negative effect of dysbiosis (Ebrahimzadeh Leylabadlo et al., 2020).

People with an increased predisposition to metabolic diseases have to be supervised for the appearance of new-onset T2D provoked by SARS-CoV-2 (Bornstein et al., 2020). As in the case of obese patients, there is a high prevalence of COVID-19 in individuals with T2D; a significant risk for increased disease severity and morbidity is also reported in such individuals (Hussain A. et al., 2020). Several factors negatively influence the predisposition of COVID-19 in patients with T2D, such as diminished viral clearance, increased cellular binding (increased ACE2 expression) and virus entry, decreased T cell function, cytokine storm syndrome and hyper-inflammation, and occurrence of cardiovascular disease (Muniyappa and Gubbi, 2020).

The unfortunate outcome in COVID-19 patients with T2D is related to increased viral entry into cells; an inhibited immune response may result in diabetic ketoacidosis and possible multi-organ failure. This type of outcome was noted in a 54-year-old man infected with SARS-CoV-2, with comorbid T2D and hypertension. Clear lungs were noted in this case, in spite of the infection (Kabashneh et al., 2020). This patient had an altered mental status, and developed seizures, diabetic ketoacidosis, and several health problems that contributed to the production of metabolic encephalopathy. After three days of adequate care and intubation, the patient recovered and exhibited an improved mental status.

In COVID-19 patients with T2D, administering insulin reduces ACE2 expression (Muniyappa and Gubbi, 2020). Therefore, physicians must be aware of the glucose status of patients with/without diabetes, and should carefully monitor all organs in diabetic patients hospitalized with COVID-19 (Kabashneh et al., 2020).

Data from 10 studies reveal a total comorbidity rate of 10.5% in 2209 Chinese patients with T2D infected with COVID-19 (Singh et al., 2020). In Italy, a higher case-fatality rate was observed, with a comorbidity rate of 35.5% among a total of 355 patients who died (Onder et al., 2020). Among 5700 COVID-19 infected patients in the New York city area, T2D was the third most frequent comorbidity (33.8%) after hypertension (56.6%) and obesity (41.7%), and these cases required invasive mechanical ventilation or ICU care more often than did patients who did not have T2D (Richardson et al., 2020). Furthermore, a higher percentage of patients with T2D evolved to acute kidney injury in comparison to patients without T2D. COVID-19 has had a negative effect on T2D, as evidenced by increased fasting blood glucose levels and deteriorated glycemic control in COVID-19 cases with T2D. These outcomes may be a result of reduced physical activity due to the imposition of quarantine, social distancing, and lockdowns, leading to changes in lifestyle (Banerjee et al., 2020). Limiting outdoor activities also reduces exposure to sunlight, leading to a vitamin D deficiency (Carter et al., 2020). This can contribute to a deranged glucose profile in diabetic patients and result in an enhanced predisposition to SARS-CoV-2 infections (Pal and Bhadada, 2020).

In summary, efficient supervision of blood glucose and careful monitoring of all major organs in worldwide patients with T2D and COVID-19 may lead to better outcomes and reduced mortality rates (Andrikopoulos and Johnson, 2020; Bornstein et al., 2020).

## Conclusions

Worldwide research on gut microbiota has increased our knowledge in the field of chronic and infectious diseases and is useful in tackling the challenges encountered in managing COVID-19 patients with associated GI diseases.

The analyzed studies indicated the bacterial potential in modulating human response to SARS-CoV-2 infection. Fecal microbiota disturbances were correlated with fecal levels of SARS-CoV-2 and COVID-19 severity. Diarrhea is a frequent presenting symptom in worldwide patients infected with SARS-CoV-2, while the increasing evidence indicates the fecal-oral transmission.

Endoscopic procedures should be done only in extreme cases and patients should be classified based on the symptoms and origin from high-risk categories. Patients with other risk factors such as age, poor diet, and comorbidities like T2D, obesity, and IBS impose a gut dysbiosis, and implicitly a severity of COVID-19 infection. Therefore, different modulation approaches to reshape the intestinal microbiota might minimize the severity of this infection and may represent a therapeutic pathway for COVID-19 co-morbidities. It is essential that current therapy approaches include a personalized diet considering that gut microbiota is modulated by diet.

As for perspectives and recommendations, future research may focus on the long-term effects of COVID-19 on the gut microbiome in order to support future actions in combating the disease at the onset of early symptoms. Further clinical investigations are needed to establish if SARS-CoV-2 induces a systemic inflammatory response via the gut epithelial passage. Revealing the possible correlation between GI symptoms and the severity of COVID-19 has major connotations for predicting disease course and establishing GI-targeted therapies that may influence disease severity.

## Author Contributions

D-CV, LM, L-FC, B-ET, KS, and G-AM researched data for the article, made a substantial contribution to the discussion of content and wrote and reviewed/edited the manuscript. LM, L-FC, B-ET, and KS made a substantial contribution to the discussion of content and wrote and reviewed/edited the manuscript. G-AM made a substantial contribution to the discussion of content and edited/reviewed the manuscript. LM, L-FC, B-ET, KS, and G-AM wrote the article. S-AN contributed to the manuscript moderate revision. All authors contributed to the article and approved the submitted version.

## Funding

The publication was supported by funds from the National Research Development Projects to finance excellence (PFE)-37/2018–2020 granted by the Romanian Ministry of Research and Innovation.

## Conflict of Interest

The authors declare that the research was conducted in the absence of any commercial or financial relationships that could be construed as a potential conflict of interest.

## Abbreviations

ACS, Acute Coronary Syndrome; ALT, Alanine Transaminase; AST, Aspartate Transaminase; GIB, Gastrointestinal Bleeding; COVID-19, Coronavirus disease; SARS-CoV-2, Severe acute respiratory syndrome coronavirus 2; WHO, World Health Organization; GI, Gastrointestinal; T2D, Type 2 Diabetes; ACE2, Angiotensin-converting enzyme 2; TNF, Tumor necrosis factor; CRP, C-reactive protein; BNP, B-type natriuretic peptide; hs-cTnI, High-sensitivity cardiac troponin I; IL, Interleukin; IBS, Irritable bowel syndrome; IBD, Inflammatory bowel disease; WOS, Web of Science; PM, PubMed.
